# The impact of an integrated depression and HIV treatment program on mental health and HIV care outcomes among people newly initiating antiretroviral therapy in Malawi

**DOI:** 10.1371/journal.pone.0231872

**Published:** 2020-05-06

**Authors:** Melissa A. Stockton, Michael Udedi, Kazione Kulisewa, Mina C. Hosseinipour, Bradley N. Gaynes, Steven M. Mphonda, Joanna Maselko, Audrey E. Pettifor, Ruth Verhey, Dixon Chibanda, Ilana Lapidos-Salaiz, Brian W. Pence

**Affiliations:** 1 Epidemiology Department, University of North Carolina at Chapel Hill Gillings School of Global Public Health, Chapel Hill, NC, United States of America; 2 NCDs & Mental Health Unit, Ministry of Health, Lilongwe, Malawi; 3 Department of Mental Health, University of Malawi, College of Medicine, Blantyre, Malawi; 4 University of North Carolina Project-Malawi, Tidziwe Centre, Lilongwe, Malawi; 5 Department of Psychiatry, University of North Carolina at Chapel Hill School of Medicine, Chapel Hill, NC, United States of America; 6 Friendship Bench Zimbabwe, Milton Park, Harare, Zimbabwe; 7 United States Agency for International Development (USAID), Arlington, VA, United States of America; University of Ghana College of Health Sciences, GHANA

## Abstract

**Background:**

Depression is highly prevalent among patients newly starting antiretroviral treatment (ART) in Malawi and many other countries. Untreated depression at ART initiation can disrupt the HIV care continuum. Effective approaches for depression screening and treatment exist for low-resource settings, but they are rarely applied. Identifying effective implementation strategies are critical.

**Methods:**

A pilot program integrated depression screening and treatment into routine HIV care using existing staff at two public health clinics in Malawi in two phases; a screening-only “control” phase and an active “intervention” phase. During the intervention phase, providers prescribed antidepressants or referred patients for Friendship Bench problem-solving therapy. We evaluated the program’s impact on retention in HIV care, viral suppression, and depression remission at 6 months using tabular comparisons and log-binomial models to estimate adjusted risk ratios and mean differences among the intervention group relative to the control group.

**Results:**

Nearly all consenting participants were screened for depression appropriately and 25% had mild to severe depressive symptoms. During the intervention phase, 86% of participants with mild depressive symptoms started Friendship Bench therapy and 96% of participants with moderate to severe depressive symptoms started antidepressants. Few participants in the intervention group received consistent depression treatment over their first 6 months in care. In the adjusted main analysis, program exposure did not demonstrably affect most HIV or mental health outcomes, though the probability of currently being on ART at 6 months was significantly lower among the intervention group than the control group [RR 0.6(95%CI: 0.4–0.9)].

**Conclusions:**

While it is feasible to integrate depression screening and treatment initiation into ART initiation, providing ongoing depression treatment over time is challenging. Similar implementation science studies focused on maintaining depression management will be increasingly important as we strive to understand and test the best ways to implement evidence-based depression treatment within HIV care.

## Introduction

The burden of depression among people living with HIV in Malawi is high, ranging between 1–19% [1–4], as in other sub-Saharan African countries [5]. Depression is associated with poor HIV care engagement and ultimately increased HIV-related mortality and morbidity [5–10]. The mechanisms through which depression undermines HIV care engagement are not fully clear. However, loss of interest, poor concentration, poor motivation, reduced self-efficacy, fatigue, hopelessness, and suicidality–key characteristics of depression–are all factors which can impair adherence and appointment attendance [8, 10, 11]. Fortunately, depression treatment programs have been developed that have the potential to improve both HIV care and mental health outcomes, with a small but growing number of interventions adapted for low- and middle-income countries [12–17]. In places such as Malawi, where nearly 10% of the adult population is living with HIV [18] and nearly a quarter of people living with HIV are lost to care in the first year of treatment [19–21], incorporating depression screening and treatment into HIV care may be key to improving engagement in care across the HIV care continuum.

There are limited resources for mental health care in Malawi, and a dearth of mental health infrastructure and specialists. Malawi treats mental healthcare as a specialized service, offered only by specialists in tertiary settings. There are only three psychiatrists and three functioning psychiatric hospitals in the country [22]. In such settings where it is unlikely that the number of specialists and infrastructure could grow rapidly enough to meet the demands of the population, task-shifting programs–models of care that shift specialized services to non-specialists [23]–are a popular, and often cost effective strategy for providing mental health services [24–26]. Of the few depression treatment interventions developed for the sub-Saharan region, most employ a task-shifting model [16, 27–32].

Two notable task-shifting interventions include algorithm-based care for depression [31–33] and the Friendship Bench behavioral activation and problem-solving therapy [29]. Algorithm-based care is a resource-efficient, task-sharing model for prescribing antidepressant management in non-psychiatric settings. This model of care has demonstrated safety, feasibility, and acceptability when adapted for HIV care and delivered by general practice medical providers in Cameroon, Tanzania and Uganda [31–33]. Developed over many years of formative research in Zimbabwe, the Friendship Bench is patient-centered counseling that teaches patients how to identify triggers and effectively manage stressful life events by learning or reactivating problem solving skills [29, 34]. These programs as well as others have proven efficacy in improving depression outcomes, though evidence on improvements in HIV care outcomes has been mixed [27, 31–33, 35–37]. Further investment in understanding the feasibility of task-shifting program implementation and the effectiveness of these models of depression care is needed to meet the mental health care needs of people living with HIV.

The Malawi Ministry of Health (MOH) has recognized the importance of addressing the burden of depression among people living with HIV as a means of improving engagement in HIV care. The MOH has also prioritized the integration of mental health services into other general health services and the development of mental health capacity of general providers [38–40]. Expanding the growing nidus of investment in mental health care programming and capacity development, the MOH implemented a pilot program integrating depression management into HIV care at two public clinics in Lilongwe, Malawi, using both algorithm-guided depression treatment and adapted Friendship Bench therapy [41]. Inspired by the key principles of implementation science, we use a multiple-baseline design to investigate the program’s impact on HIV care and depression outcomes.

## Methods

### Objectives

The main objective of this study was to evaluate the program’s impact on retention, viral suppression, and depression remission among patients with elevated depressive symptoms at antiretroviral therapy (ART) initiation after 6 months in care.

### Study design

**Fig 1:**
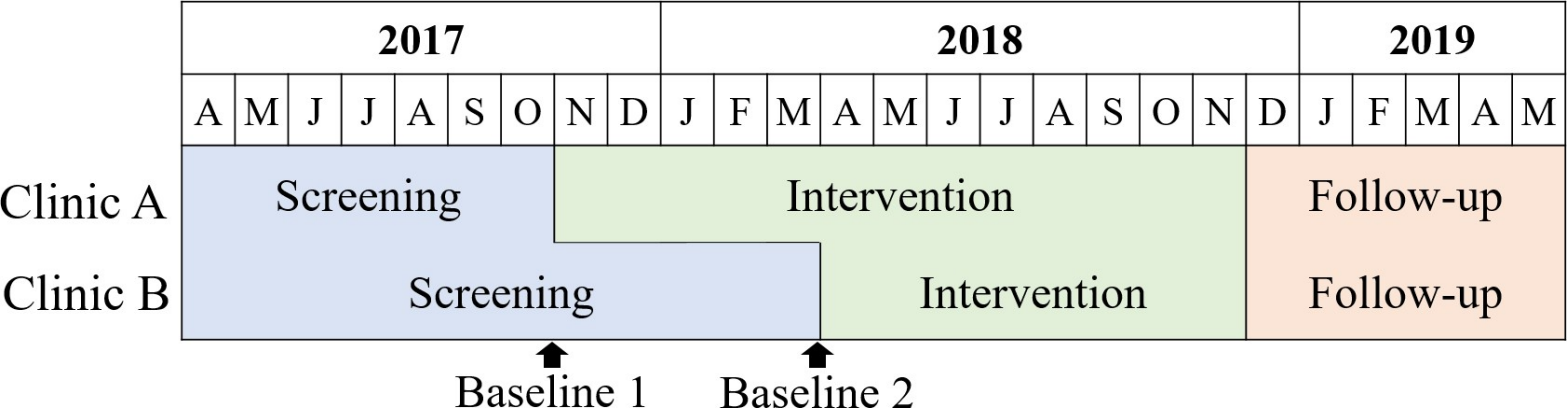
Staggered multiple baseline study design.

This study employed a staggered multiple baseline (pre-post) design to evaluate a pilot program integrating depression screening and treatment into routine HIV primary care using existing staff at two public health clinics in Lilongwe, Malawi [42]. Multiple baseline studies use a time-series design that can be used for studies with multiple sites in which each site intentionally receives the intervention at a different time point [42, 43]. This design can also provide evidence of causal relationships where randomization is not possible and provides stronger control for temporal trends [42, 43]. As such, the program rolled out in two phases, a screening-only “control” phase and an active “intervention” phase. The screening control phase launched at both clinics in April 2017. However, the launch of the intervention phase was staggered, launching at Clinic A in November 2017 and at Clinic B in April 2018. (**Fig 1)** Additional information on the study design is available in the published protocol paper [41].

Control phase:

During the control phase, providers screened patients for depression using the Patient Health Questionnaire-9 (PHQ-9). The PHQ-9, a nine-item screening questionnaire that assesses the presence and frequency of the nine Diagnostic and Statistical Manual of Mental Disorders, Fourth Edition symptoms of major depression [44]. It has been widely used in the region [2, 45, 46] and was recently validated in Malawi among patients with diabetes [47]. A total score of 5–9 and ≥10 are considered indicative of mild depressive symptoms and moderate to severe depressive symptoms, respectively[48]. Patients who endorsed the last question of the PHQ-9, which asks about thoughts of being better off dead or hurting oneself, completed a suicide risk assessment protocol (SRAP) with the providers. The SRAP guided providers’ evaluation of whether such thoughts were passive or active. During the control phase, ART providers were re-oriented to existing options for depression care within the Malawi primary care system. This options theoretically include counseling and antidepressants, although in actuality counseling consists of informal counseling by ART providers and antidepressants are rarely prescribed. For patients with suicidality, providers were trained to respond based on the level of severity, ranging from a brief safety assessment to immediate transport to the outpatient psychiatric department at the district hospital.

During the intervention phase, ART providers (nurses or clinicians) used PHQ-9 scores at ART initiation to triage patients by depressive severity. ART providers were trained to refer participants with mild depressive symptoms (PHQ-9 score 5–9) to clinic-based lay health workers or a project-employed counselor trained to provide an adapted problem-solving intervention called Friendship Bench therapy [27, 29]. These counselors provided patient-centered counseling that guided patients through recognizing problems, identifying their own solutions, implementing those solutions, and assessing the outcome[29, 34]. Patients referred to the Friendship Bench would ideally receive at least six counseling sessions. While these patients were encouraged to return weekly for Friendship Bench therapy, patients set their own appointments in line with the protocol’s patient-centered approach. For participants with moderate to severe depressive symptoms (PHQ-9 score ≥10), ART providers were trained to prescribe antidepressants (fluoxetine or amitriptyline) with appropriate dosing and dose changes, using an algorithm-based care protocol. Under this protocol, providers used PHQ-9 scores and drug side effects to guide antidepressant prescription, titrating the antidepressant dose by monitoring depressive symptoms and antidepressants side effects at each clinic visit [31–33] (**Table 1)**. Patients who started antidepressants were meant to be reevaluated monthly at their ART visits when they would be re-prescribed antidepressants for at least three months. Additional information on the combined medication and counseling depression treatment program has previously been published [22].

**Table 1:** Depression treatment overview, by phase.

Score	Depressive Severity	Screening “Control” Phase	Active “Intervention” Phase
0–4	No or minimal	None	None
5–9	Mild	No formal depression treatment–could include informal clinician counseling or, rarely, antidepressant prescription	Friendship Bench
10–27	Moderate to severe		Antidepressants

### Study population

From April 2017 through November 2018, all non-pregnant adults newly initiating ART at the study sites were eligible to be included in the program evaluation. Pregnant women were excluded as they typically test for HIV at a specific antenatal care clinic and continue to receive ART as part of their antenatal care. This analysis is restricted to participants who completed depression screening and had elevated depressive symptoms (PHQ-9 score ≥ five) at ART initiation. Participants were placed in the “control” or “intervention” groups based on the phase during which they initiated ART.

Control group: Includes participants with elevated depressive symptoms who initiated ART at either clinic during the screening “control” phase (e.g. at Clinic A between April 2017 and November 2017 and at Clinic B between April 2017 and April 2018).

Intervention group: Includes participants with elevated depressive symptoms who initiated ART at either clinic after the launch of the treatment program during the active “intervention” phase (e.g. at Clinic A after November 2017 and at Clinic B after April 2018).

### Data collection

As an implementation science study, the primary objective was to assess the impact of an intervention that could readily (and rapidly) be adopted and integrated into routine care in the public sector setting [49, 50]. Just as the program made use of existing clinical staff and systems, the evaluation was intentionally designed to influence and disrupt the provision of care as little as possible by collecting only data that was already routinely captured during clinical care and using research staff only to consent patients and abstract data. Specifically, we did not conduct baseline interviews or schedule six-month follow-up interviews, which would have limited enrollment and potentially brought participants back into care, biasing our primary outcome measure of HIV care engagement. Research assistants at the study sites approached potential participants during ART initiation to invite them to allow their clinical data to be used in the program evaluation. Research assistants abstracted routinely collected clinic data on depression and HIV care from consenting participants’ clinical records over a 13-month period, starting at ART initiation. Abstracted data included appointment dates, expected return dates, ART pills dispensed, PHQ-9 scores, and depression treatment provided. At ART initiation and each subsequent ART appointment, providers give patients a follow-up appointment date and a sufficient supply of ART. Generally, for the first six months of care, new ART patients receive a 30-day supply of ART and a return appointment date in 30 days at each appointment. Most often, patients need to attend monthly ART refill appointments for their first six months of care in order to maintain their ART supply. Additionally, we obtained electronic medical record data from the clinical sites to ensure the quality and completeness of the abstracted data.

### Measures

#### Exposures

Adequate Friendship Bench therapy should consist of six sessions over the first six months in care and a standard course of antidepressants should consist of at least three consecutive months of antidepressant prescription with dosage adjustments as necessary. For the analysis purposes, we have operationalized the intervention exposure as follows:

Intent-to-treat: Intervention group participants were considered “exposed” to the intervention if they initiated ART during the intervention phase. Control group participants were considered “unexposed” to the intervention if they initiated ART during the control phase.

Treatment started: The intervention group included participants who either had: 1) mild depressive symptoms at ART initiation who started Friendship Bench therapy at ART initiation or 2) moderate to severe depressive symptoms who were prescribed antidepressants at ART initiation. The control group participants did not start Friendship Bench therapy or antidepressants at ART initiation.

As treated: The intervention group included participants who attended at least their first follow-up appointment and either had: 1) mild depressive symptoms at ART initiation and at least two Friendship Bench therapy sessions over their first six months in care or 2) moderate to severe depressive symptoms at ART initiation and were prescribed at least two months of antidepressants over their first six months in care. The control group included participants who attended at least their first follow-up appointment, but did not have at least two friendship bench sessions or two months of antidepressant prescription over their first six months in care. Those participants who did not attend at least their first follow-up visit were excluded. While this definition was less stringent than the ideal course of treatment, this definition allowed for a more refined comparison of those who received early and consistent depression treatment compared to those who had not.

#### Outcomes

Retention was defined as never being more that 14 days late to an appointment through 6 months in care. Maintaining a consistent ART supply was defined as never more than 5 days without ART in the first 6 months, calculated from the cumulative days’ supply of ART dispensed at each appointment in the first 6 months and the time between appointments. In alignment with the PEPFAR definition of “currently on treatment” [51], participants were currently on ART 6 months after ART initiation if they attended an appointment and received a supply of ART that would last through the 6 month mark. Viral suppression was defined as a viral load of less than 1,000 copies/mL drawn after at least five and a half months (166 days) in care. Depression remission was defined as a PHQ-9 score of less than five taken after at least five and a half months (166 days) in care, to allow for completion of the acute phase of treatment and maintenance of that response [52]. Additionally, we examined the proportion of scheduled HIV appointments attended within one week during the first 6 months of care and the ART pill possession ratio, the proportion of the first 6 months (183 days) since ART initiation with ART in hand, calculated from cumulative pills dispensed at each ART appointment through 6 months.

#### Covariates

Due to the implementation science nature of this study, we only abstracted routinely collected clinical data. Measured covariates included the healthcare facility where patients received ART (Clinic A or B), months since program launch at ART initiation, gender, age, baseline depressive severity at ART initiation (mild or moderate to severe), and baseline presence of suicidal thoughts at ART initiation. Presence of suicidal thoughts was defined as any endorsement of the ninth question of the PHQ-9, “During the past two weeks, how many days have you been bothered by thoughts that you would be better off dead or of hurting yourself in some way?”

### Data analysis

The main analysis followed an “intent-to-treat” approach, classifying participants according to screening “control” vs. active “intervention” phase (unexposed vs. exposed to the program) without regard to actual treatment received. Although patients identified with elevated depressive symptoms late in the control phase could theoretically have received depression care from providers during the intervention phase during their first six months of care, in practical terms, depression treatment was only provided to patients newly entering care. Furthermore, we monitored crossover between groups. We first completed unadjusted, tabular comparisons of HIV care and depression outcomes in the intervention group relative to the control group. We then used log-binomial models to estimate adjusted risk ratios comparing the probability of the primary outcome, retention with viral suppression, as well as the probability of other secondary HIV care outcomes in the intervention group compared to the control group. Since the evaluation employed a staggered multiple baseline design, with the intervention launching at different dates at the two clinics, all models were adjusted for clinic and months since program launch to allow for the assessment of and potential correction for any secular trend in outcomes (“confounding by history”). While the study design should have produced comparable control and intervention groups, we did additionally consider controlling for measured covariates. To separately evaluate the impact of the Friendship Bench and the antidepressant treatment, we then stratified the primary analysis by depressive severity at baseline (mild or moderate to severe depressive symptoms). Other secondary analyses used a “treatment started” approach, comparing those who actually started Friendship Bench therapy or antidepressants to those that did not in both phases and then an “as treated” approach, comparing those who received at least two Friendship Bench therapy sessions or two months of antidepressants to patients who did not in both phases, restricted to only those who attended at least their first follow-up visit.

Outcome data for those who transferred or died in the first 6-months of care were treated as missing. To address missing outcome data, we used multiple imputation by chained equations (MICE) to fill missing values using logistic regression imputation methods [53]. We imputed values based on phase, healthcare facility, months since program launch at ART initiation, age, sex, baseline depressive severity at ART initiation and baseline presence of suicidality at ART initiation. We generated 18 imputed datasets to ensure the number of imputed datasets was at least as large as the percentage of incomplete information for the main outcome, “retention with viral suppression” [54]. To verify that this was sufficient, we confirmed that the number of imputed datasets was also larger than the parameter-specific fraction of missing information for all parameters included in the final model [55].

All analyses were performed using STATA IC 14.

### Ethical review

The National Health Sciences Research Committee of Malawi (NHSRC) and the Biomedical Institutional Review Board (IRB) of the University of North Carolina at Chapel Hill approved the study protocol. All participants gave written informed consent to allow the abstraction of their clinical data. The evaluation only used de-identified clinic data to ensure the protection of participants’ identities and confidentiality. Patients with depression received depression care regardless of whether or not they provided consent for their data to be abstracted.

## Results

### Depression screening

**Fig 2:**
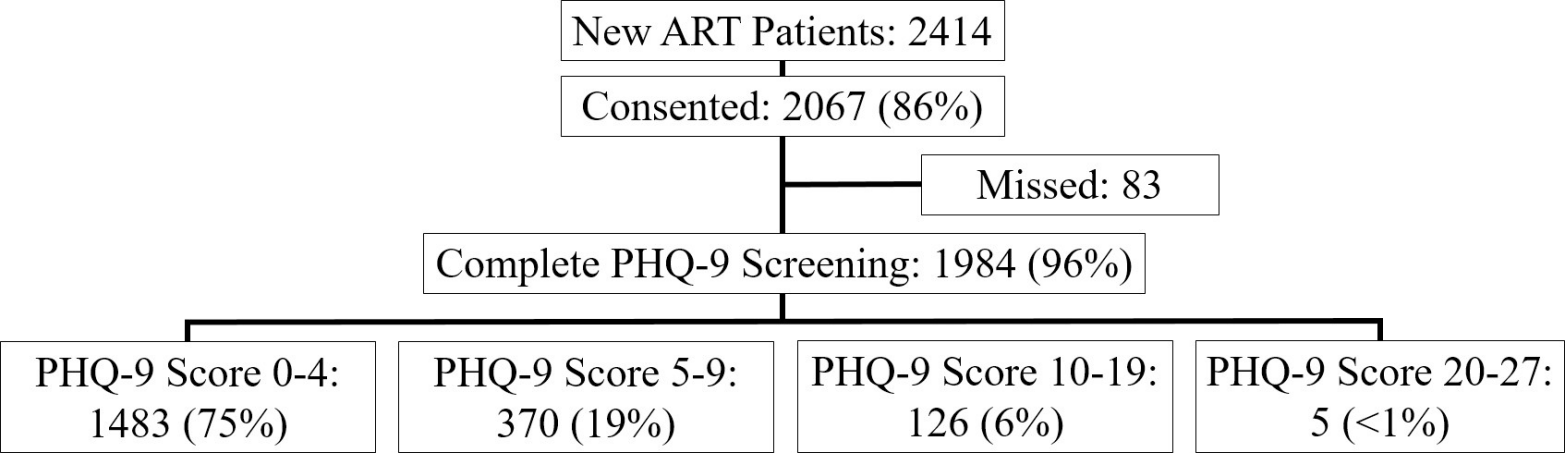
Evaluation enrollment.

Over the course of the program, 2414 patients newly initiated ART (**Fig 2)**. Of these new initiators, 2067 (86%) consented to allow their data to be abstracted to evaluate the program. Nearly all of those who consented (96%) were screened with the PHQ-9 appropriately. Among those who completed the PHQ-9 screening, the prevalence of mild depressive symptoms (PHQ-9 score 5–9) was 19% and the prevalence of moderate to severe depressive symptoms (PHQ-9 score ≥ 10) was 6%. Only those participants with elevated depressive symptoms (PHQ-9 score ≥ 5) are included in this analysis (N = 501).

### Participant characteristics

Of the 501 consenting participants with elevated depressive symptoms, the control group consisted of the 290 who initiated ART during the control phase and the intervention group consisted of the 211 who initiated during the intervention phase (**Table 2)**. As the program launched later at Clinic B, a larger proportion (75%) of the intervention group were from Clinic A. Otherwise, the two groups appear very similar. About 43% of participants were male. The average age of participants was 33.8 years. Nearly all participants were classified as asymptomatic (Stage I) for HIV at ART initiation, according to the World Health Organization’s (WHO) HIV clinical stages for HIV surveillance [56]. At ART initiation, 74% of participants had mild depressive symptoms, only around 1% of participants had severe depressive symptoms and 21% of participants reported suicidality.

**Table 2:** Participant characteristics (N = 501).

n(%) or mean (sd)	Overall	Control Group	Intervention Group
Overall	501	290	211
Clinic			
Clinic A	276 (55%)	116 (40%)	160 (76%)
Clinic B	225 (45%)	174 (60%)	51 (24%)
Sex			
Male	214 (43%)	125 (43%)	89 (42%)
Female	287 (57%)	165 (57%)	122 (58%)
Age	33.8 (9.5)	33.6 (10.0)	34.0 (8.7)
WHO Disease Stage			
I	498 (99%)	288 (99%)	210 (>99%)
II-IV	3 (1%)	2 (1%)	1 (<1%)
Baseline Depression Severity			
Mild (PHQ-9: 5–9)	370 (74%)	214 (74%)	156 (74%)
Moderate (PHQ-9: 10–19)	126 (25%)	71 (24%)	55 (26%)
Severe (PHQ-9: 20–27)	5 (1%)	5 (2%)	0 (0%)
Baseline Suicidality			
No thoughts	397 (79%)	228 (79%)	169 (80%)
Passive thoughts	53 (11%)	33 (11%)	20 (9%)
Active thoughts	46 (9%)	27 (9%)	19 (9%)
Not assessed with SRAP	5 (1%)	2 (1%)	3 (1%)

SRAP = Suicide Risk Assessment Protocol

### Depression treatment exposure

#### Initiating depression treatment

Among the control group, 89% of participants with mild depressive symptoms and 80% participants with moderate to severe depressive symptoms received some additional counseling by ART clinicians (“standard depression care” during the control phase) (Table 3). During this phase, only 18% (n = 14) of those with moderate to severe depressive symptoms started amitriptyline during the control phase and most (n = 11) started at a subtherapeutic dose of 25 mg.

**Table 3:** Depression treatment initiation.

N (%)	Control Group	Intervention Group
**Mild depressive symptoms (PHQ-9: 5–9)**	214 (100%)	156 (100%)
Counseling by clinician	191 (89%)	15 (10%)
Friendship Bench therapy	-	134 (86%)
Antidepressant*	2 (1%)	2 (1%)
None**	21 (10%)	5 (3%)
**Moderate to severe depressive symptoms (PHQ-9: ≥10)**	76 (100%)	55 (100%)
Counseling by clinician	60 (79%)	2 (4%)
Friendship Bench therapy	-	0 (0%)
Antidepressant***	14 (18%)	53 (96%)
None	2 (3%)	0 (0%)

*Includes those who received both antidepressants and the Friendship Bench (n = 2).

**Those with missing baseline treatment plan (n = 4) treated as none.

***Includes those who received both antidepressants and counseling by clinician (n = 8) or both antidepressants and the Friendship Bench (n = 4).

Most of the intervention group started the appropriate treatment as defined by the intervention protocol during the intervention phase. During this phase, 86% of participants with mild depressive symptoms started Friendship Bench therapy and nearly all (96%) participants with moderate to severe depressive symptoms started antidepressants. All of the participants with moderate to severe depressive symptoms who started antidepressants at ART initiation started at a minimal effective dose (operationally defined as a daily oral dose of either 20 mg of fluoxetine or 50 mg of amitriptyline).

#### Provision of ongoing depression treatment

Few patients in the intervention group continued to receive ongoing depression treatment during the intervention phase after ART initiation. Only 42% of participants referred to the Friendship Bench attended three or more therapy sessions (Table 4). Of those who were prescribed antidepressants, less than a third of participants (n = 17) received three or more months of antidepressant prescription. Only six of those participants received at least three consecutive months of the same antidepressant. Inadequate sustained treatment was in part due to poor appointment attendance, medication stock-outs, and clinicians’ failure to identify patients with elevated depressive symptoms at follow-up ART appointments. However, it did not appear that there was much crossover between groups. None of the control participants ever started Friendship Bench therapy and very few started antidepressants at ART initiation (n = 16) or at a follow-up visit (n = 6).

**Table 4:** Depression treatment over time.

	Control Group	Intervention Group
Of those directed to Friendship Bench:*	-	140 (100%)
Number of sessions attended		
1	-	60 (43%)
2	-	21 (15%)
≥3	-	59 (42%)
Of those prescribed antidepressants:**	16 (100%)	55 (100%)
Number of months antidepressants provided		
1	13 (81%)	23 (42%)
2	2 (13%)	15 (27%)
≥3	1 (6%)	17 (31%)

*Includes those who were referred to the Friendship Bench and those who were both prescribed antidepressants and referred to the Friendship Bench.

**Includes those who were prescribed antidepressants and those who were both prescribed antidepressants and were referred to the Friendship Bench.

### Program impact

Most HIV and mental health outcomes did not appear to differ between groups. (**Table 5)**. Retention in both arms was poor; approximately a third of participants in both groups remained in care through 6 months. Just over half of the control group and just under half of the intervention group were currently on ART at six months. Close to 40% of participants in both the control and intervention phases maintained a consistent ART supply (never more than 5 days without ART) through six months. Many participants had missing data for viral suppression and depression remission after 5.5 months; only 36% (n = 181) of participants had viral load draw after 5.5 months and 26% of participants (n = 132) had a measured PHQ-9 score. Among those with a measured viral load or PHQ-9 score after 5.5 months, respectively, viral suppression and depression remission were high (>91%) in both groups. Findings were similar when stratified by baseline depressive severity (mild or moderate to severe) and when using a “treatment started” and an “as treated” approach. **S1 Table–S4 Table**.

**Table 5:** Program impact on HIV and depression outcomes.

n(%) or mean (sd)	Control	Intervention
Retention: never >14 days through 6 months	91/266 (34%)	66/186 (35%)
HIV appointment attendance: average proportion of scheduled appointments attended through 6 months (Range: 0–1)	0.57 (0.39)	0.59 (0.38)
Currently on ART: attended appointment prior to 6 months with next scheduled appointment after 6 months	138/266 (52%)	87/186 (47%)
Consistent ART: never >5 days without ART through 6 months	112/266 (42%)	73/186 (39%)
ART pill possession: average proportion of days with ART through 6 months (Range: 0.16–1)	0.69 (0.35)	0.67 (0.37)
Viral suppression: VL < 1,000 copies/mL after 5.5 months, among those with a viral load	108/115 (94%)	60/66 (91%)
Depression remission: PHQ-9 score < 5 after 5.5 months, among those with a PHQ-9 score	87/93 (94%)	37/38 (97%)

Transferred within the first 6 months of care: Control Phase n = 24; Intervention Phase n = 25; Denominators vary due to viral loads not being drawn, the PHQ-9 not being administered, not having or attending a scheduled appointment around 6 months.

To assess potential bias due to missing data from transferring or attending care without getting a 6-month viral load or PHQ-9 assessment, we compared baseline information between those with and without missing data. While a larger proportion of transfers were from Clinic A, missing data did not appear to be associated with any other measured baseline characteristics. **S5 Table–S7 Table**.

The final adjusted model controlled for clinic, months since program launch at ART initiation, sex and baseline depressive severity. Both age and baseline presence of suicidality introduced model instability. As age did not appear to be associated with any of the outcomes or program exposure, we ultimately removed it from the model. We only retained baseline depressive severity in the final adjustment set as baseline presence of suicidality was highly correlated with baseline depressive severity.

In the adjusted “intent-to-treat” analysis, program exposure did not demonstrably affect the primary outcome, retention with viral suppression at 6 months (**Table 6).** While no difference was evident for most of the secondary outcomes, the probability of currently being on ART at 6 months and the ART pill possession ratio was significantly lower among the intervention group than among the control group [RR 0.6(95%CI: 0.4–0.9) and Mean Difference -0.1(95%CI: -0.3–0), respectively]. Correction for missing data with multiple imputation did not have a significant impact on any of the outcomes. When stratified by baseline severity, only the probability of being on ART at 6 months was significantly lower during the intervention phase than during the control phase [RR 0.33(95%CI: 0.13–0.83)] among those with moderate to severe depressive symptoms at baseline. **S8 Table.** The “treatment started” and “as treated” approaches generally showed similar results. **S9 Table and S10 Table.**

**Table 6:** Effect of intervention on HIV care and depression outcomes.

Outcome	Adjusted*	Imputation**
	RR or Mean Difference (95%CI)	
Retention: never >14 days through 6 months	1.1 (0.6–1.9)	1.1 (0.6–1.9)
HIV appointment attendance: average proportion of scheduled appointments attended through 6 months	0.0 (-0.1–0.2)	0.0 (-0.1–0.1)
Currently on ART: attended appointment prior to 6 months with next scheduled appointment after 6 months	**0.6 (0.4–0.9)**	**0.6 (0.4–0.9)**
Consistent ART: never >5 days without ART through 6 months	0.8 (0.5–1.2)	0.8 (0.5–1.3)
ART pill possession: average proportion of days with ART through 6 months (Range: 0.16–1)	**-0.1 (-0.3–0.0)**	-0.1 (-0.4–0.1)

*Adjusted for clinic, months since program launch (quadratic term), sex, and baseline depressive severity

**Further corrected for missing data via multiple imputation.

## Discussion

We investigated the impact of a program integrating depression management into HIV care initiation on 6-month HIV care and depression outcomes. Clinic staff screened nearly all patients for depression, documenting the prevalence of mild and moderate to severe depressive symptoms among people newly initiating ART. During the intervention phase of the program, they successfully started the majority of the intervention group on the appropriate depression treatment. However, providing ongoing treatment proved more challenging, and few patients received a standard course of antidepressants or attended a sufficient number of Friendship Bench therapy sessions. Retention was very low in both the intervention and control groups. Nearly all participants who did remain in care and had a 6-month viral load drawn and PHQ-9 assessment achieved viral suppression and depression remission. However, the evaluation did not yield evidence that the integrated depression treatment program improved 6-month HIV care or depression outcomes among the intervention group compared to the control group.

The successful integration of depression screening allowed this evaluation to document the prevalence of depression among people newly initiating ART. A growing body of research is beginning to establish the magnitude of mental health disorders in Malawi. In Malawian primary care settings, a third of all patients have a common mental disorder, most commonly depression [57]. However, few studies have estimated the prevalence of depression among the general adult population of people living with HIV. We found that around a quarter of people newly initiating ART at the study sites had mild to severe depressive symptoms. This finding is only slightly higher than estimates from various subpopulations (adolescents, pregnant women, adults receiving HIV care) of people living with HIV in Malawi [1–3] and supports other sub-Saharan regional estimates of depression prevalence [5]. Such evidence indicates a clear need for depression treatment among people living with HIV.

The integration of depression screening and treatment initiation at the time of ART initiation appeared feasible. During ART initiation, nearly all of the 2067 participants who consented to participate in the study were successfully screened for depression. Further, nearly all intervention participants started the treatment appropriate for their depressive severity at ART initiation. Factors that may have contributed to this success include the effective utilization of existing ART initiation processes and the creation of a collaborative training environment [22]. For example, after careful study of patient flow, we designed the initial PHQ-9 screening to be shared by the HTC counselors and the ART providers, distributing the additional work and time burden. We also ensured that every HIV provider received training in how to administer and interpret the PHQ-9, provide depression treatment, and manage depression symptoms over time–creating opportunities for iterative learning and ongoing support [22]. Another task-sharing study in Uganda found that both depression screening by lay workers and antidepressant treatment initiation by ART provider were feasible, a success the researchers attributed to ongoing training and appropriate mentorship [33]. As such, using non-psychiatric specialists to screen and triage cases of depressions appears possible in this sub-Saharan setting.

While effective integration into existing processes was key to the success of depression screening and treatment initiation, the aspects of existing infrastructure and supply management that the program was unable to effectively utilize hampered the provision of sustained depression treatment. Providers at the study sites typically rely on an electronic medical records (EMR) system to provide ART, which did not incorporate PHQ-9 screening or depression treatment and thus did not alert providers to re-assess depressed patients returning for care. Despite developing a system of marking patients’ health passports so that those needing depression treatment re-evaluation could be identified, providers struggled to re-identify depressed patients returning for ART care[22]. Very few studies on task-shifting models of care in the region have relied on existing staff (as opposed to study employed staff) or assessed the provision of depression treatment over time. For example, the study evaluating different task-sharing strategies for depression treatment in Uganda only reported on depression treatment initiation [33], making comparisons challenging. Other implementation science studies will need to track and assess the provision of treatment over time.

Antidepressant stock-outs were also common and problematic. While the Malawi Ministry of Health has committed to making antidepressants freely available at health center pharmacies [40], ensuring their availability was complicated and required substantial coordination, often beyond the scope of work of the health center pharmacist. Other countries such as Mozambique with similar policies on the provision of antidepressants have also experienced challenges stocking these medications at the district or clinic level [58]. Another task-shifting depression program in Tanzania also experienced anti-depressant stock-outs [31]. Greater engagement and investment of health sector stakeholders involved in the procurement, supply and distribution of drugs such as Malawi’s Central Medical Stores is required to ensure antidepressant stocks are maintained to ensure the success and safety of depression treatment programs.

The clinics also found it challenging to provide proper Friendship Bench therapy, in part due to community health care workers’ availability and in part due to patients’ ability to return to the clinic for therapy sessions, in light of financial, time, and transport barriers[59]. As the community health workers already had a very high workload and often traveled off-site to run various Ministry of Health initiatives, the one program-employed counselor at each site ultimately provided the majority of the therapy sessions. Community health workers are often already overloaded with work and are at risk of becoming additionally overburdened when drawn into task-sharing models of care [60, 61], as was potentially the case at the study sites. Furthermore, while the original Friendship Bench protocol called for 6 weeks of weekly therapy sessions [29], patients were reluctant to make additional trips to the clinic primarily due to time and transportation barriers. This resulted in scheduling Friendship Bench sessions to coincide with the patients’ monthly ART refill appointments. While attending weekly sessions appeared acceptable in the original Friendship Bench program [29], it is difficult to tell from other adaptations as they may have incentivized attending sessions with reimbursement [62]. These findings highlight the importance of implementation science research to establish the real-world feasibility of task-shifting depression care for people living with HIV.

As implemented, the program did not appear to improve HIV care or depression outcomes, as few patients received depression treatment as intended. Studies in the region have found that similar programs, albeit with higher fidelity to their treatment protocols, have been effective at treating depression [35, 36]. In our study, nearly all participants achieved depression remission, regardless of depression treatment. As many of these other evaluations of depression treatment programs were quite small or did not include control arms [35, 36], it is possible that depressive symptoms could have improved in the absence of depression treatment. However, it should be noted that we could have overestimated six-month depression remission if patients who did not remain in care were more likely to have persistent depressive symptoms. In regards to the program’s impact on HIV outcomes, the literature on linking depression treatment to improvements across the HIV care cascade is mixed [13–16, 62–69]. A recent meta-analysis of depression treatment interventions for people living with HIV in sub-Saharan Africa also did not find that interventions improve viral suppression, though they did conclude that the programs with an ART adherence component had the greatest impact on HIV outcomes [36]. As it was impossible for the program to have a pure control group in which patients were denied depression treatment, it is possible that providers discussed the depression screening results with participants or provided brief additional ART adherence counseling. While even unstructured additional patient attention has the potential to impact engagement in care, it is no substitute for evidence-based depression treatment and adherence counseling interventions. Further research is needed to understand the factors that influenced adherence to the program protocol and to identify the more effective ways to implement depression treatment programs that will improve depression and HIV care outcomes.

Retention in care was remarkably low in both of the study phases; only a quarter of patients achieved retention and viral suppression at 6 months. Aligned with PEPFAR’s less stringent definition of being in care at 6 months (our “currently on ART” measure) [51], only half of patients were retained at six months. These estimates are much lower than national 12-month estimates from 2018, which found that 65% of adults who initiated ART were still in care at 12 months[70]. However, as our entire study population had elevated depressive symptoms, it possible that depression is responsible for this difference in retention. The program did not appear to improve HIV care engagement, even when using the “treatment started” or “as treated” approach, and in the main analysis the intervention group was significantly less likely to currently be on ART at 6-months than the control group. It is possible that this population faced other barriers to care that were more relevant and difficult to overcome, such as human resource and institutional challenges, distance to the clinic, lack of support, stigma and fear of HIV status disclosure [71–74]. Nonetheless, there is a distinct need for urgent action to improve early retention in care for people living with HIV and depression.

### Limitations

The results should be considered in light of several limitations. Although the study’s design was not guided by a formal implementation science framework, the study was designed in congruence with the key principles of implementation science, using methods that would promote the systematic uptake of evidence-based practices (e.g. the Friendship Bench and algorithm-based care) into routine care [75, 76]. In line with the implementation science-inspired design of the study and our efforts not to unduly influence the provision of or engagement in care, all of the measures were drawn from routinely collected medical chart data. As we did not conduct baseline interviews or schedule six-month follow-up interviews, we captured limited information on potential confounders. A large proportion of patients did not have six-month viral loads or PHQ-9 scores due to either dropout or provider failure to refer patients for viral load testing or screen patients appropriately. Still, the multiple baseline design should have protected against confounding from unmeasured confounders by producing balanced characteristics across phases, as evident from similar characteristics of intervention and control groups seen in **Table 2**. Additionally, the program struggled to establish a sense of ownership among providers, develop capacity to manage depression treatment over time, and ensure the availability of antidepressants, which may have impacted providers’ adherence to the depression treatment protocol [22]. As an extension of this challenge, ART providers administered the six-month PHQ-9, so part of the observed reduction in PHQ-9 scores could be due to less careful administration.

All of the HIV engagement measures may have been influenced by participants who sought care at another facility without formally transferring their records. These “silent transfers” would have been misclassified as being out of care. However, as the rate of silent transfers should not have varied between groups, this potential measurement error should not have introduced bias into the analyses.

## Conclusion

This experience demonstrated that while it is feasible to integrate depression screening and treatment initiation into ART initiation, providing ongoing depression treatment over time is challenging; very few patients received ongoing depression treatment for many of the reasons discussed above. In this setting, it is clear that in order for such a program to be successful additional resources are needed to support ongoing capacity building, ensure the availability of Friendship Bench counselors and antidepressants, encourage patient engagement in the full-course of treatment, and further integrate into the electronic medical records system. Additionally, further research is needed to explore the other factors that potentially govern both the implementation of and engagement with such a program. While the evaluation did not yield evidence that the program improved HIV care or depression outcomes in a real world clinic setting, depression treatment in sub-Saharan Africa is efficacious for improving mental health and engagement in HIV care in more controlled research environments [35]. Further, this program targeted patients newly initiating ART, often at the highest risk for loss to care. Further research could explore whether other patient populations may benefit from depression treatment, such as those returning for care with elevated depressive symptoms or whether depression treatment for people living with HIV may be more effective if it includes a specific component supporting ART adherence or HIV care engagement. Moving the field of mental health care in LMICs forward, similar implementation science studies will be increasingly important as we strive to understand and test the best ways to implement evidence-based depression treatment protocols for this vulnerable population.

## Supporting information

S1 Table Program impact on HIV and depression outcomes, among those with mild depression (N = 371). (DOCX)

S2 Table Program impact on HIV and depression outcomes, among those with moderate to severe depression (N = 131). (DOCX)

S3 Table Program impact on HIV and depression outcomes, “treatment started” approach* (N = 502). (DOCX)

S4 Table Program impact on HIV and depression outcomes, “as treated” approach* (N = 358). (DOCX)

S5 Table Participant characteristics, by transfer (N = 502). (DOCX)

S6 Table Participant characteristics, by viral load data (N = 249). (DOCX)

S7 Table Participant characteristics, by 6-month PHQ-9 data (N = 241). (DOCX)

S8 Table Association between depression treatment and HIV care and depression outcomes, by depressive severity at baseline. (DOCX)

S9 Table Association between depression treatment and HIV care and depression outcomes, “treatment started” approach*. (DOCX)

S10 Table Association between depression treatment and HIV care and depression outcomes, “as treated” approach*. (DOCX)
