# Supplementary material for: The impact of an integrated depression and HIV treatment program on mental health and HIV care outcomes among people newly initiating antiretroviral therapy in Malawi
Source: PLoS One. 2020 May 6;15(5):e0231872. doi: 10.1371/journal.pone.0231872 (PMC7202614; doi:10.1371/journal.pone.0231872)
Supplement: S1 Table — (DOCX) [file pone.0231872.s001.docx]

**S1 Table: Program impact on HIV and depression outcomes, among those with mild depressive symptoms (N=370)**

| n(%) or mean(sd) | **Screening Phase**  **Control** | **Active Phase**  **Intervention** |
| --- | --- | --- |
| Retention: never >14 days through 6 months | 68/199 (34%) | 49/136 (36%) |
| HIV appointment attendance: average proportion of scheduled  appointments attended through 6 months (Range: 0-1) | 0.6 (0.4) | 0.6 (0.4) |
| Currently on ART: attended appointment prior to 6 months  with next scheduled appointment after 6 months | 106/199 (53%) | 66/136 (49%) |
| Consistent ART: never >5 days without ART through 6 months | 85/199 (43%) | 55/136 (40%) |
| ART pill possession: average proportion of days with ART  through 6 months (Range: 0.16-1) | 0.7 (0.4) | 0.7 (0.4) |
| Viral suppression: VL < 1,000 copies/mL after 5.5 months,  among those with a viral load | 83/88 (94%) | 45/49 (92%) |
| Depression remission: PHQ-9 score < 5 after 5.5 months, among  those with a PHQ-9 score | 63/67 (94%) | 27/28 (96%) |

Transferred within the first 6 months of care: Control Phase n=15; Intervention Phase n=20; Denominators vary due to viral loads not being drawn, the PHQ-9 not being administers, not having or attending a scheduled appointment around 6 months.
