# Supplementary material for: The impact of an integrated depression and HIV treatment program on mental health and HIV care outcomes among people newly initiating antiretroviral therapy in Malawi
Source: PLoS One. 2020 May 6;15(5):e0231872. doi: 10.1371/journal.pone.0231872 (PMC7202614; doi:10.1371/journal.pone.0231872)
Supplement: S3 Table — (DOCX) [file pone.0231872.s003.docx]

**S3 Table: Program impact on HIV and depression outcomes, “treatment started” approach* (N=501)**

| n(%) or mean(sd) | **Untreated** | **Started Treatment** |
| --- | --- | --- |
| Retention: never >14 days through 6 months | 88/269 (33%) | 69/183 (38%) |
| HIV appointment attendance: average proportion of scheduled  appointments attended through 6 months (Range: 0-1) | 0.6 (0) | 0.6 (0) |
| Currently on ART: attended appointment prior to 6 months  with next scheduled appointment after 6 months | 135/269 (50%) | 90/183 (49%) |
| Consistent ART: never >5 days without ART through 6 months | 108/269 (40%) | 77/183 (42%) |
| ART pill possession: average proportion of days with ART  through 6 months (Range: 0.16-1) | 0.7 (0.4) | 0.7 (0.4) |
| Viral suppression: VL < 1,000 copies/mL after 5.5 months,  among those with a viral load | 103/109 (94%) | 65/72 (90%) |
| Depression remission: PHQ-9 score < 5 after 5.5 months, among  those with a PHQ-9 score | 82/87 (94%) | 42/44 (95%) |

*“Treatment started” approach compares patients who started the Friendship Bench or antidepressants to patients who did not start either; Transferred within the first 6 months of care: Untreated n=27; Started Treatment n=22; Denominators vary due to viral loads not being drawn, the PHQ-9 not being administers, not having or attending a scheduled appointment around 6 months.
