# Supplementary material for: The impact of an integrated depression and HIV treatment program on mental health and HIV care outcomes among people newly initiating antiretroviral therapy in Malawi
Source: PLoS One. 2020 May 6;15(5):e0231872. doi: 10.1371/journal.pone.0231872 (PMC7202614; doi:10.1371/journal.pone.0231872)
Supplement: S4 Table — (DOCX) [file pone.0231872.s004.docx]

**S4 Table: Program impact on HIV and depression outcomes, “As treated” approach* (N=355)**

| n(%) or mean(sd) | **Untreated** | **Started Treatment** |
| --- | --- | --- |
| Retention: never >14 days through 6 months | 125/268 (47%) | 32/54 (59%) |
| HIV appointment attendance: average proportion of scheduled  appointments attended through 6 months (Range: 0-1) | 0.8 (0) | 0.8 (0) |
| Currently on ART: attended appointment prior to 6 months  with next scheduled appointment after 6 months | 168/268 (62%) | 38/54 (70%) |
| Consistent ART: never >5 days without ART through 6 months | 137/268 (51%) | 32/54 (59%) |
| ART pill possession: average proportion of days with ART  through 6 months (Range: 0.16-1) | 0.8 (0.3) | 0.9 (0.2) |
| Viral suppression: VL < 1,000 copies/mL after 5.5 months,  among those with a viral load | 126/134 (95%) | 25/29 (86%) |
| Depression remission: PHQ-9 score < 5 after 5.5 months, among  those with a PHQ-9 score | 96/103 (93%) | 22/22 (100%) |

*“As treated” approach compares patients who received at least two Friendship Bench therapy sessions or their first two months antidepressants to patients who did not, restricted to only those who attended at least their first follow-up visit; Transferred within the first 6 months of care: Inadequate n=25; Adequate n=8; Denominators vary due to viral loads not being drawn, the PHQ-9 not being administers, not having or attending a scheduled appointment around 6 months.
