# Supplementary material for: The impact of an integrated depression and HIV treatment program on mental health and HIV care outcomes among people newly initiating antiretroviral therapy in Malawi
Source: PLoS One. 2020 May 6;15(5):e0231872. doi: 10.1371/journal.pone.0231872 (PMC7202614; doi:10.1371/journal.pone.0231872)
Supplement: S5 Table — (DOCX) [file pone.0231872.s005.docx]

**S5 Table: Participant characteristics, by transfer (N=501)**

| n(%) or mean(sd) | **Overall** | **Didn’t Transfer** | **Transferred** |
| --- | --- | --- | --- |
| Overall | 501 | 452 | 49 |
| Clinic |  |  |  |
| Clinic A | 276 (55%) | 239 (53%) | 37 (76%) |
| Clinic B | 225 (45%) | 213 (47%) | 12 (24%) |
| Sex |  |  |  |
| Male | 214 (43%) | 193 (43%) | 21 (43%) |
| Female | 287 (57%) | 259 (57%) | 28 (57%) |
| Age | 33.8 (9.5) | 33.9 (9.5) | 33.2 (8.9) |
| Baseline Depression Severity |  |  |  |
| Mild (PHQ-9: 5-9) | 370 (74%) | 335 (74%) | 35 (71%) |
| Moderate to severe (PHQ-9: 10-27) | 131 (26%) | 117 (26%) | 14 (29%) |
| Baseline Suicidality |  |  |  |
| No thoughts | 397 (79%) | 354 (78%) | 43 (88%) |
| Suicidal thoughts | 104 (21%) | 98 (22%) | 6 (12%) |
