# Supplementary material for: The impact of an integrated depression and HIV treatment program on mental health and HIV care outcomes among people newly initiating antiretroviral therapy in Malawi
Source: PLoS One. 2020 May 6;15(5):e0231872. doi: 10.1371/journal.pone.0231872 (PMC7202614; doi:10.1371/journal.pone.0231872)
Supplement: S6 Table — (DOCX) [file pone.0231872.s006.docx]

**S6 Table: Participant characteristics, by viral load data (N=255)**

| n(%) or mean (sd) | **Overall** | **Viral Load** | **Attended, but no Viral Load** |
| --- | --- | --- | --- |
| Overall | 255 | 181 | 74 |
| Clinic |  |  |  |
| Clinic A | 148 (59%) | 109 (60%) | 39 (53%) |
| Clinic B | 107 (41%) | 72 (40%) | 35 (47%) |
| Sex |  |  |  |
| Male | 110 (43%) | 70 (39%) | 40 (54%) |
| Female | 145 (57%) | 111 (61%) | 34 (46%) |
| Age | 33.8 (9.5) | 35.3 (9.8) | 34.9 (9.2) |
| Baseline Depression Severity |  |  |  |
| Mild (PHQ-9: 5-9) | 189 (74%) | 137 (76%) | 52 (70%) |
| Moderate to severe (PHQ-9: 10-27) | 66 (26%) | 44 (24%) | 22 (30%) |
| Baseline Suicidality |  |  |  |
| No thoughts | 195 (76%) | 133 (73%) | 62 (84%) |
| Suicidal thoughts | 60 (24%) | 48 (27%) | 12 (16%) |
