# Supplementary material for: The impact of an integrated depression and HIV treatment program on mental health and HIV care outcomes among people newly initiating antiretroviral therapy in Malawi
Source: PLoS One. 2020 May 6;15(5):e0231872. doi: 10.1371/journal.pone.0231872 (PMC7202614; doi:10.1371/journal.pone.0231872)
Supplement: S7 Table — (DOCX) [file pone.0231872.s007.docx]

**S7 Table: Participant characteristics, by 6-month PHQ-9 data (N=241)**

| n(%) or mean (sd) | **Overall** | **PHQ-9** | **Attended, but no PHQ-9** |
| --- | --- | --- | --- |
| Overall | 241 | 131 | 110 |
| Clinic |  |  |  |
| Clinic A | 143 (59%) | 80 (61%) | 63 (57%) |
| Clinic B | 98 (41%) | 51 (39%) | 47 (42%) |
| Sex |  |  |  |
| Male | 104 (43%) | 53 (40%) | 51 (46%) |
| Female | 137 (57%) | 78 (60%) | 589(54%) |
| Age | 33.8 (9.5) | 35.2 (10.2) | 35.2 (8.9) |
| Baseline Depression Severity |  |  |  |
| Mild (PHQ-9: 5-9) | 178 (74%) | 95 (73%) | 83 (75%) |
| Moderate to severe (PHQ-9: 10-27) | 63 (26%) | 36 (27%) | 27 (25%) |
| Baseline Suicidality |  |  |  |
| No thoughts | 185 (77%) | 96 (73%) | 89 (81%) |
| Suicidal thoughts | 56 (23%) | 35 (27%) | 21 (19%) |
