# Supplementary material for: The impact of an integrated depression and HIV treatment program on mental health and HIV care outcomes among people newly initiating antiretroviral therapy in Malawi
Source: PLoS One. 2020 May 6;15(5):e0231872. doi: 10.1371/journal.pone.0231872 (PMC7202614; doi:10.1371/journal.pone.0231872)
Supplement: S8 Table — (DOCX) [file pone.0231872.s008.docx]

**S8 Table: Association between depression treatment and HIV care and depression outcomes, by depressive severity at baseline**

|  | **Mild** | **Moderate to Severe** |
| --- | --- | --- |
|  | aRR or Mean Difference (95%CI) | |
| Retention: never >14 days through 6 months | 1.2 (0.6-2.3) | 0.8 (0.3-2.4) |
| HIV appointment attendance: average proportion of  scheduled appointments attended through 6 months | 0.0 (-0.1-0.2) | -0.1 (-0.4-0.2) |
| Currently on ART: attended appointment prior to 6 months  with next scheduled appointment after 6 months | 0.7 (0.5-1.1) | **0.3 (0.1-0.8)** |
| Consistent ART: never >5 days without ART through 6  months | 0.8 (0.5-1.4) |  |
| ART pill possession: average proportion of days with ART  through 6 months | -0.1 (-0.3-0.0) | -0.2 (-0.5-0.1) |

*Adjusted for clinic, months since program launch (quadratic term) and sex.
