# Supplementary material for: The impact of an integrated depression and HIV treatment program on mental health and HIV care outcomes among people newly initiating antiretroviral therapy in Malawi
Source: PLoS One. 2020 May 6;15(5):e0231872. doi: 10.1371/journal.pone.0231872 (PMC7202614; doi:10.1371/journal.pone.0231872)
Supplement: S9 Table — (DOCX) [file pone.0231872.s009.docx]

**S9 Table: Association between depression treatment and HIV care and depression outcomes, “treatment started” approach***

| Outcome | **Adjusted**** | **Imputation***** |
| --- | --- | --- |
|  | RR or Mean Difference (95%CI) | |
| Retention: never >14 days through 6 months | 1.4 (0.9-2.1) | 1.4 (0.9-2.1) |
| HIV appointment attendance: average proportion of  scheduled appointments attended through 6 months | 0.1 (0.0-0.2) | 0.0 (-0.1-0.1) |
| Currently on ART: attended appointment prior to 6 months  with next scheduled appointment after 6 months | 1.0 (0.8-1.3) | 1.0 (0.8-1.3) |
| Consistent ART: never >5 days without ART through 6  months | 1.2 (0.8-1.7) | 1.2 (0.8-1.7) |
| ART pill possession: average proportion of days with ART  through 6 months | 0.0 (-0.1-0.1) | 0.0 (-0.1-0.1) |

*“Treatment started” approach compares patients who started the Friendship Bench or antidepressants to patients who did not start either; **Adjusted for clinic, months since program launch (quadratic term), sex, and baseline depressive severity; ***Pooled estimates from imputed datasets.
