# Supplementary material for: The impact of an integrated depression and HIV treatment program on mental health and HIV care outcomes among people newly initiating antiretroviral therapy in Malawi
Source: PLoS One. 2020 May 6;15(5):e0231872. doi: 10.1371/journal.pone.0231872 (PMC7202614; doi:10.1371/journal.pone.0231872)
Supplement: S10 Table — (DOCX) [file pone.0231872.s010.docx]

**S10 Table: Association between depression treatment and HIV care and depression outcomes, “as treated” approach***

| Outcome | **Adjusted**** | **Imputation***** |
| --- | --- | --- |
|  | RR or Mean Difference (95%CI) | |
| Retention: never >14 days through 6 months | 1.3 (1.0-1.9) | 1.4 (1.0-2.0) |
| HIV appointment attendance: average proportion of  scheduled appointments attended through 6 months | 0.1 (0.0-0.1) | **0.3 (0.2-0.4)** |
| Currently on ART: attended appointment prior to 6 months  with next scheduled appointment after 6 months | 1.1 (0.9-1.4) | 1.2 (0.9-1.5) |
| Consistent ART: never >5 days without ART through 6  months | 1.1 (0.8-1.5) | 1.1 (0.8-1.6) |
| ART pill possession: average proportion of days with ART  through 6 months | 0.1 (0.0-0.2) | 0.1 (0.0-0.1) |

*“As treated” approach compares patients who received at least two Friendship Bench therapy sessions or their first two months antidepressants to patients who did not, restricted to only those who attended at least their first follow-up visit; **Adjusted for clinic, months since program launch (quadratic term), sex, and baseline depressive severity; ***Pooled estimates from imputed datasets.
